# Targeted Panel Sequencing Identifies an Intronic c.5225-3C>G Variant of the *FBN1* Gene Causing Sporadic Marfan Syndrome with Annuloaortic Ectasia

**DOI:** 10.3390/genes13112108

**Published:** 2022-11-13

**Authors:** Kyung Hwa Kim, Tae Yun Kim, Soon Jin Kim, Yong Gon Cho, Joonhong Park, Woori Jang

**Affiliations:** 1 Department of Thoracic and Cardiovascular Surgery, Jeonbuk National University Medical School and Hospital, Jeonju 54907, Korea; 2 Department of Laboratory Medicine, Jeonbuk National University Medical School and Hospital, Jeonju 54907, Korea; 3 Research Institute of Clinical Medicine of Jeonbuk National University-Biomedical Research Institute of Jeonbuk National University Hospital, Jeonju 54907, Korea; 4 Department of Laboratory Medicine, College of Medicine, Inha University, Incheon 22232, Korea

**Keywords:** targeted panel sequencing, intronic variant, c.5225-3C>G, *FBN1* gene, Marfan syndrome, annuloaortic ectasia, aortic valve regurgitation

## Abstract

Marfan syndrome (MFS) is a hereditary connective tissue disease whose clinical severity varies widely. Mutations of the *FBN1* gene encoding fibrillin-1 are the most common genetic cause of Marfanoid habitus; however, about 10% of MFS patients are unaware of their genetic defects. Herein, we report a Korean patient with MFS and annuloaortic ectasia caused by an intronic c.5225-3C>G variant of the *FBN1* gene identified by targeted panel sequencing. The reverse transcription analysis of *FBN1* revealed that the intron 43 sequence from positions c.5297-1516 to c.5297-1 was retained at the coding sequence as a consequence of the c.5225-3C>G variant enhancing a cryptic splice acceptor site (c.5297-1518_5297-1517AG) in intron 43. The retained sequence of the part of intron 43 caused the same effect as insertion mutation (NM_000138.5:c.5297_c.5298ins5297-1516_5297-1), resulting in a frameshift mutation resulting in p.Ile1767Trpfs*3. The patient underwent an urgent modified Bentall operation with a 29 mm mechanical valve for annuloaortic ectasia and severe aortic valve regurgitation. This report emphasizes the need for functional investigations into the diagnostic workflows of certain diseases or gene panels with suspected high rates of intronic variants and potential pathogenic effects. Hence, further descriptions of individuals with intronic variants causing alternative splicing expected to have pathogenic effects at different transcript levels are crucial for improving our understanding.

## 1. Introduction

Marfan syndrome (MFS, OMIM #154700) is a hereditary connective tissue disease whose clinical severity varies widely from isolated characteristics to neonatal presentations of rapidly progressive and severe diseases involving multiple organ systems [1,2,3]. Marfan syndrome occurs globally without geographic, ethnic, or professional predispositions and affects both males and females equally. Its incidence in the general population has been estimated to be 2–3 per 10,000 individuals [4]. In Korea, the age-standardized overall prevalence of MFS in 2013 was 2.27 persons (1.92 in females and 2.61 in males) per 100,000 persons [5]. The diagnosis of MFS is based on the evaluation of many clinical criteria. The clinical features of MFS overlap with those of other connective tissue disorders and are collectively considered under the definition of Marfanoid habitus [6]. The complexity of the clinical manifestations of MFS and its unpredictable progression have been investigated extensively over the past few decades, especially in an attempt to explain the genotype–phenotype correlations of Marfanoid habitus and MFS. Mutations of the *FBN1* gene encoding fibrillin-1 are the most common genetic cause of Marfanoid habitus, and *FBN1* mutations can be detected in >90% of individuals with classic MFS [7]. Previous studies that aimed to study the genotype–phenotype correlations in Marfanoid habitus focused mainly on the pathogenic variants of *FBN1* [8,9]. More than 3000 causative mutations of *FBN1* have been reported, and the mutation spectrum consists of missense, frameshift, and nonsense mutations as well as exon deletions [10]. Among them, most (> 90%) of the *FBN1* mutations are located in the coding region and at specific splice sites. *FBN1* mutations have often been classified according to their domain localization or as in-frame mutations or protein-truncating types without considering the effects on the protein product [11,12]. However, about 10% of patients with MFS are unaware of their genetic defects [4]. Because additional genes are associated with Marfanoid habitus [2,4,13,14], the *FBN1* gene cannot be considered a unique genetic cause of Marfanoid habitus. Further, the clinical significances of some variants, such as intronic variants that may affect splicing, are uncertain and cannot be interpreted appropriately, despite the development of next-generation sequencing (NGS) technologies [15,16].

Cases of Koreans with MFS carrying the *FBN1* mutations have been reported [17,18,19,20,21,22], and a few cases caused by non-splice-site intronic variants have been identified [18]. Herein, we report a Korean patient with MFS and annuloaortic ectasia caused by an intronic c.5225-3C>G variant of the *FBN1* gene identified by targeted panel sequencing; in vitro characterization of the effects of this variant on complementary DNA (cDNA) showed that it was a pathogenic variant.

## 2. Materials and Methods

### 2.1. Targeted Panel Sequencing

Genomic DNA was extracted from the whole blood of the proband and their parents using a QIAamp DNA Mini Kit (Qiagen GmbH, Hilden, Germany). Singleton targeted panel sequencing using Celemics’ G-Mendeliome clinical exome sequencing panel (Celemics, Seoul, Korea) was performed on NextSeq500 instrument (Illumina, San Diego, CA, USA) with a high output flow cell and 300 Paired-end cycles (150 × 2) at the Green Cross Genome (Yongin, Korea) to detect variants, given the suspicion of hereditary aortopathy and connective tissue disorder. In particular, the coding region and adjacent intronic sequences of 22 genes, including *ACTA2*, *BGN*, *CBS*, *COL1A1*, *COL1A2*, *COL3A1*, *COL5A1*, *COL5A2*, *FBN1*, *FBN2*, *LOX*, *MFAP5*, *MYH11*, *MYLK*, *NOTCH1*, *PRKG1*, *SKI*, *SMAD3*, *TGFB2*, *TGFB3*, *TGFBR1*, and *TGFBR2,* were selected as described elsewhere [14,23]. Base calling, alignment, variant calling, annotation, and quality control reporting were performed using the Genome Analysis Tool Kit best-practice pipeline workflow for germline short variant discovery (https://gatk.broadinstitute.org/hc/en-us, accessed on 18 April 2022). Deoxyribonucleic acid sequencing reads were aligned with the human genome reference assembly GRCh38 (hg38) using the Burrows–Wheeler aligner (BWA). As a result, the targeted panel sequencing generated 1,288,546,132 target read samples via estimation of the sequence quality along all sequences. The mean read depth (×) was 108, and the percentage of bases above a read depth of 30× was 93.3%. The interpretation of the sequence variants was reviewed manually by medical laboratory geneticists according to the standards and guidelines of the Joint Consensus Recommendation of the American College of Medical Genetics and Genomics (ACMG) and Association for Molecular Pathology (AMP) for classifying the pathogenic variants [24]. Briefly, the filtering scheme used to find the potential causative variant is as follows: (1) variants located within or near exons of protein-coding genes; (2) variants with allele frequencies < 0.01; (3) variants causing nonsense or nonsynonymous changes in codons within exons, causing frameshift mutations, or altering the highly conserved splice sites; (4) homozygous or compound heterozygous variants of the same gene or de novo variants identified in the proband only. The disorder is most likely considered to be autosomal recessive or sporadic inheritance because the proband’s parents were unaffected.

### 2.2. Verification of the FBN1 c.5225-3C>G Variant

Sanger sequencing of the polymerase chain reaction (PCR) products was conducted using the BigDye Terminator v3.1 Cycle Sequencing Kit (Applied Biosystems, Foster City, CA, USA) and was resolved by capillary electrophoresis on a 3730XL Genetic Analyzer (Applied Biosystems, Carlsbad, CA, USA). The presence of the *FBN1* c.5225-3C>G variant was confirmed with bidirectional Sanger sequencing using the primer pairs 5′–AGGCACTCTTACCAGTCCCT–3′ (NG_008805.2:g.185137_185156) and 5′–ATTAGGTGGAGCTGCACAGG–3′ (NG_008805.2:g.185705_185724). The functional impact on alternative splicing was predicted using the Human Splicing Finder (HSF, https://hsf.genomnis.com/, accessed on 21 May 2022) [25] and SpliceAI (https://spliceailookup.broadinstitute.org/, accessed on 21 May 2022) [26]. Variant allelic frequency was assessed using public genome databases, the genome aggregation database (gnomAD, https://gnomad.broadinstitute.org/, accessed on 24 May 2022), and the Korean reference genome database (KRGDB, https://coda.nih.go.kr/coda/KRGDB/, accessed on 28 May 2022). ClinVar (https://www.ncbi.nlm.nih.gov/clinvar/, accessed on 28 May 2022), a public database of variant interpretations, was used to identify if any candidate variants were previously reported as (likely) pathogenic.

### 2.3. Ribonucleic Acid Isolation, Reverse Transcription PCR, and Complementary DNA Sequencing

Total RNA was extracted from the peripheral blood of the proband as well as healthy controls using a QIAGEN miRNeasy Micro Kit (Qiagen, Hilden, Germany) following manufacturer protocols. The cDNA was prepared with a QuantiTect Reverse Transcription Kit (Qiagen) and used as a template in subsequent PCRs for cDNA analysis. The alternative splicing effect of the c.5225-3C>G variant on *FBN1* was analyzed by reverse transcription PCR (RT-PCR) amplification, and the RT-PCR products were separated by 2% agarose/tris-acetate-EDTA buffer gel electrophoresis. The visible bands were excised, extracted from gel pieces using a GeneJET PCR Purification Kit (Thermo Scientific, Waltham, MA, USA), and Sanger sequenced using the primer pairs 5′-TGGAATCTGTGGTCCAGGGA-3′ (NM_000138.5:c.4965_4984) and 5′-ATTGCACTGTCCTGTGGAGG-3′ (NM_000138.5:c.5525_5544) including exons 41 through 45 of the *FBN1* gene.

## 3. Results

**Figure 1:**
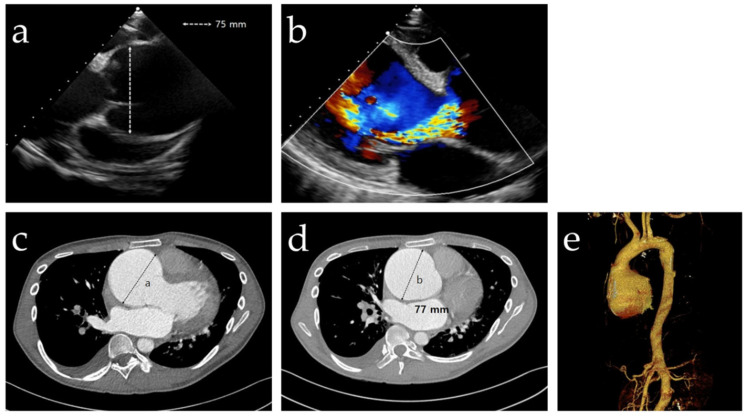
Radiologic findings of the proband. (**a**,**b**) Transthoracic echocardiography revealed a valsalva sinus aneurysm (dotted arrow) with severe aortic valve regurgitation. (**c**,**d**) The computed tomographic aortogram (CTA) showed a valsalva sinus aneurysm and proximal ascending aortic aneurysm in the trans-sectional images. (**e**) The three-dimensional reconstructed image of the CTA revealed annuloaortic ectasia.

A 23-year-old male patient, the firstborn male child of healthy Korean parents at 39 weeks of gestation, visited the emergency room of Jeonbuk National University Hospital (Jeonju, Korea) through a local clinic because of gradually worsening dyspnea for two weeks. He had been undergoing outpatient follow-up at our Department of Orthopedic Surgery for scoliosis for eight years and had no medical history of other diseases. There were no specific findings in the family history. He was 185 cm tall and weighed 63 kg, with a slender build. He had arachnodactyly, but the thumb sign was negative. Myopia was observed, but the predominant ocular complication of MFS, such as ectopia lentis or lens subluxation, was not found. Transthoracic echocardiography (TTE) was performed to evaluate the dyspnea and revealed severe aortic valve regurgitation (AR) with aortic annular dilatation. The size of the annulus in the TTE was 75 mm. The left ventricular (LV) ejection fraction was 29%, showing severe LV systolic dysfunction. The LV end-diastolic and end-systolic dimensions were 88 and 79 mm, respectively (Figure 1a,b). A large aneurysm was detected via computed tomographic aortogram in the valsalva sinus and proximal ascending aorta, termed annuloaortic ectasia, and the maximum diameter of the ascending aorta was 77 mm (Figure 1c–e). The patient underwent an emergency modified Bentall operation with a 29 mm mechanical valve for the annuloaortic ectasia and severe AR.

**Figure 2:**
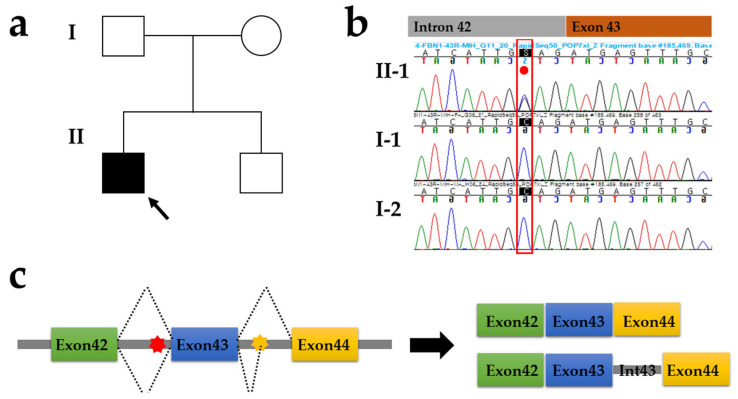
Pedigree analysis and Sanger sequencing analysis. (**a**) Pedigrees of the proband with sporadic Marfan syndrome and annuloaortic ectasia as well as his family members. (**b**) Sanger sequencing confirmed a heterozygous intronic c.5225-3C>G variant of the *FBN1* gene (red dot) in the proband only. (**c**) Intron retention, in which an intron remains in the mature mRNA transcript rather than being removed during maturation caused by c.5225-3C>G variant of the *FBN1* gene (red star). Yellow star, a cryptic splice acceptor site (c.5297-1518_5297-1517AG) in intron 43.

Targeted panel sequencing was performed to determine the potential genetic cause of hereditary aortopathy and connective tissue disorder in the proband only (II-1 in Figure 2a). A heterozygous intronic c.5225-3C>G variant in the *FBN1* gene was identified as the best candidate for the genetic cause of MFS in the proband. The c.5225-3C>G substitution was located three nucleotides upstream of the intron 42/exon 43 junction of the *FBN1* gene (Figure 2b). The c.5225-3C>G of intron 42 was predicted to have caused a break in the wild-type (WT) acceptor site, with a delta value of −92.77% (score for WT: 9.27; score for variant: 0.67), which most probably affected splicing, as determined by MaxEnt generated from the HSF. Similarly, SpliceAI predicted that this intronic splice variant might lead to acceptor loss with a score of 0.94 and pre-mRNA position of −3 bp without donor loss, acceptor gain, or donor gain as SpliceAI recommended prioritizing variants as potentially splice-altering if the cutoff was greater than 0.2.

**Figure 3:**
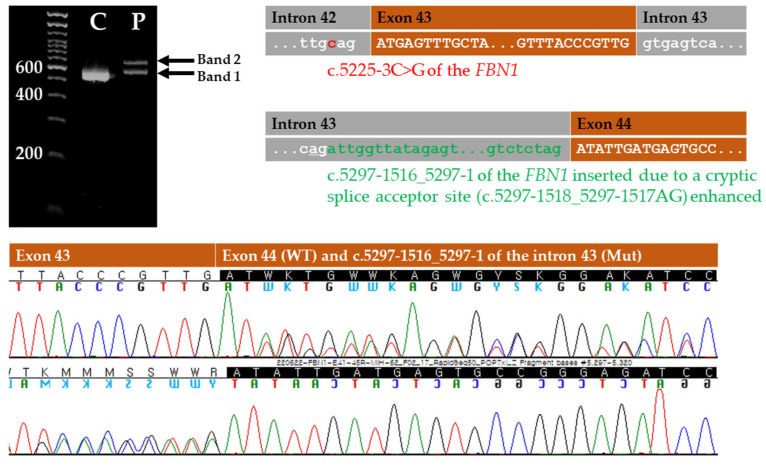
Results of complementary DNA sequencing of the *FBN1* c.5225-3C>G variant causing alternative acceptor site splicing. Reverse transcription PCR of *FBN1* transcripts between exons 41 and 45 in the proband demonstrated that a cryptic splice acceptor site (c.5297-1518_5297-1517AG) in intron 43 (underlined) was enhanced and the retained sequence of the part of intron 43 caused the same effect as insertion mutation (NM_000138.5:c.5297_c.5298ins5297-1516_5297-1), resulting in a frameshift mutation resulting in p.Ile1767Trpfs*3. C, normal control. P, proband.

The RT-PCR analyses of the *FBN1* transcripts in whole blood from the proband and control using a primer set between exons 41 and 45 demonstrated that the intron 43 sequence from positions c.5297-1516 to c.5297-1 was retained at the coding sequence as a consequence of the c.5225-3C>G variant enhancing a cryptic splice acceptor site (c.5297-1518_5297-1517AG) in intron 43 (Figure 2c). The retained sequence of the part of intron 43 caused the same effect as insertion mutation (NM_000138.5:c.5297_c.5298ins5297-1516_5297-1), resulting in a frameshift mutation resulting in p.Ile1767Trpfs*3 (Figure 3). The cDNA sequencing supported the damaging effects of this rare *FBN1* variant, which was classified as PVS1 and defined as a null variant (nonsense, frameshift, canonical ±1 or 2 splice sites, initiation codon, single- or multi-exon deletion) in a gene where the loss of function (LoF) is a known mechanism of disease based on ACMG-AMP criteria.

The proband’s family members presented no clinical symptoms associated with MFS, and genetic counseling and segregation analysis were performed to identify the genetic origin of MFS in the patient. Sanger sequencing confirmed that the unaffected parents were genetically normal. Thus, a de novo intronic variant c.5225-3C>G in the *FBN1* gene was identified to cause sporadic MFS in the proband.

## 4. Discussion

Several large cohorts with autosomal dominant inherited MFS and related diseases have been studied previously, and a definitive molecular diagnosis was reported in 40–95% of them depending on the testing strategy and inclusion criteria [11,27,28]. Currently, genetic approaches are preferred for testing individuals with MFS and Marfanoid habitus using a gene panel rather than single-gene analysis, followed by multiplex ligation-dependent probe amplification for negative samples because of the relevant number of mutations affecting genes other than *FBN1* [29]. Particularly, targeted panel sequencing that includes a broader list of candidate genes as well as a better and more thorough evaluation of the clinical manifestations of MFS and Marfanoid habitus may improve genetic diagnoses considerably [13,14,23]. For example, Nayak et al. obtained a higher molecular diagnosis rate of 85% in 45 of 53 MFS families using targeted multiple-gene panels, whole-exome sequencing, and multiplex ligation-dependent probe amplification [23]. Furthermore, the implementation of targeted panel sequencing in individuals with hereditary aortopathies or hereditary connective tissue disorders in a clinical setting has been proven to be useful for achieving genetic diagnoses [30,31,32]. Thus, targeted panel sequencing is the most practical screening strategy for identifying disease-causing variants in individuals with the clinical features of typical MFS and Marfanoid habitus.

The type of *FBN1* mutation detected and its likelihood of being pathogenic are considered important factors in the diagnosis of MFS and for later clinical decisions. For example, a high frequency of splicing and truncating *FBN1* mutations was observed in patients with Ghent-positive MFS along with aortic dissection and/or surgery compared to patients with *FBN1*-mutation-positive MFS without reported aortic events [10]. In support of this finding, our report describes a Korean patient with MFS and annuloaortic ectasia as well as severe AR caused by a novel intronic *FBN1* variant; the patient received an emergency modified Bentall operation with a 29 mm mechanical valve. We confirmed the effects of this variant enhancing a cryptic splice acceptor site (c.5297-1518_5297-1517AG) along with a normal splice acceptor site (c.5297-2_5297-1AG) and added this novel variant to the *FBN1* mutational repertoire. This variant caused intron retention, in which an intron remains in the mature mRNA transcript rather than being removed during maturation. Ribonucleic acid sequencing and other data demonstrate that intron retention is a common mechanism of tumor-suppressor inactivation, is widespread across cancer entities, and contributes to their transcriptional diversity [33]. This was an LoF variant and expected to result in either a loss of protein or an abnormal truncated protein product from the mutant allele through nonsense-mediated mRNA decay. Thus, the c.5225-3C>G variant was classified as pathogenic according to the revised Ghent nosology for MFS. In this case, we highlighted the significance of including extended intronic regions to interpret NGS results for variant prioritization and postanalytical work for medical purposes. This case also emphasizes the requirement to investigate the functional effects to further verify the pathogenicity of intronic variants with potentially damaging effects. This novel intronic c.5225-3C>G variant of *FBN1* has not been reported previously as benign or pathogenic and has not been observed in the general population (gnomAD) or Korean population (KRGDB). The different nucleotide substitution was one of two variant alleles of a single nucleotide and registered as rs876657810 (NM_000138.5:c.5225-3C>A) in the Single Nucleotide Polymorphism database (dbSNP; https://www.ncbi.nlm.nih.gov/snp/, accessed on 17 June 2022), even though it was reported in ClinVar as having “uncertain significance” (https://www.ncbi.nlm.nih.gov/clinvar/variation/228685/?new_evidence=false, accessed on 20 October 2022). Similar to our novel intronic variant, the c.5225-3C>A variant was predicted to cause a break in the WT acceptor site, with a delta of −45.2% (score for WT: 9.27; score for variant: 5.08) by MaxEnt, leading to an acceptor loss with a score of 0.72 and pre-mRNA position of −3 bp by SpliceAI. In particular, it is important to investigate noncoding intronic variants that are not located on canonical splice sites at the RNA level before classification. Splicing analysis should be preferably carried out using RNA from peripheral blood samples. However, in most cases, RNA is not available from the patient. Alternatively, the variant can be examined by mini-gene splicing analysis, which has been described previously to be a valid method for investigating the impacts of variants on the splicing patterns [34,35]. These findings are expected to have important implications for the management of patients with MFS having *FBN1* splicing and truncating variants, especially in light of the fact that the current practices generally consider such patients to have milder disease progression.

Advances in NGS technologies in molecular diagnostics have improved the costs and turnaround times, but they have not significantly influenced the clinical interpretations of uncertain significance variants, particularly intronic nucleotide substitutions, which potentially affect alternative splicing. Some previous case reports [16,36,37,38] as well as the present case suggest the impacts of variants on the noncoding DNA in MFS and related disorders, in addition to the opportunity to reconsider the standard diagnostic approaches in certain cases [38]. The burden related to uncertain results associated with these variants is still high in MFS as the point variants possibly impacting *FBN1* pre-mRNA splicing seem to account for up to 10% of the genetic findings reported for MFS [39]. Thus, optimal clinical interpretation in these cases should integrate genetic findings with customized studies exploring the variant effects at the transcriptional or translational levels. Such an approach also necessitates the availability of additional validated diagnostic tests and the ability to explore variant effects at the mRNA or post-transcriptional levels to support clinical interpretations of the genomic data. These variants also need to be verified for disease-causing effects based on public sequence databases, such as the Leiden Open Variation Database (https://www.lovd.nl/), ClinVar (https://www.ncbi.nlm.nih.gov/clinvar/), and Human Gene Mutation Database (https://www.hgmd.cf.ac.uk/) [40].

## 5. Conclusions

In conclusion, we report a novel heterozygous *FBN1* c.5225-3C>G variant causing alternative acceptor site splicing, thereby resulting in sporadic MFS. This finding expands the clinical and pathological spectra of the genotype–phenotype correlations associated with MFS caused by *FBN1* intronic variants. This report also emphasizes the need for functional investigations into the diagnostic workflows of certain diseases or gene panels with suspected high rates of intronic variants and potential pathogenic effects. Further descriptions of individuals with intronic variants causing alternative splicing and expected to have pathogenic effects at different transcript levels are crucial for improving our understanding of MFS.

## Data Availability

Not applicable.

## References

[1] Stark V.C., Hensen F., Kutsche K., Kortüm F., Olfe J., Wiegand P., von Kodolitsch Y., Kozlik-Feldmann R., Müller G.C., Mir T.S. (2020). Genotype-Phenotype Correlation in Children: The Impact of FBN1 Variants on Pediatric Marfan Care. Genes.

[2] Arnaud P., Milleron O., Hanna N., Ropers J., Ouali N.O., Affoune A., Langeois M., Eliahou L., Arnoult F., Renard P. (2021). Clinical relevance of genotype-phenotype correlations beyond vascular events in a cohort study of 1500 Marfan syndrome patients with FBN1 pathogenic variants. Genet. Med..

[3] Hernándiz A., Zúñiga A., Valera F., Domingo D., Ontoria-Oviedo I., Marí J.F., Román J.A., Calvo I., Insa B., Gómez R. (2021). Genotype FBN1/phenotype relationship in a cohort of patients with Marfan syndrome. Clin. Genet..

[4] Verstraeten A., Alaerts M., Van Laer L., Loeys B. (2016). Marfan Syndrome and Related Disorders: 25 Years of Gene Discovery. Hum. Mutat..

[5] Jang S.Y., Seo S.R., Park S.W., Kim D.K. (2017). The Prevalence of Marfan Syndrome in Korea. J. Korean Med. Sci..

[6] Rybczynski M., Bernhardt A.M., Rehder U., Fuisting B., Meiss L., Voss U., Habermann C., Detter C., Robinson P.N., Arslan-Kirchner M. (2008). The spectrum of syndromes and manifestations in individuals screened for suspected Marfan syndrome. Am. J. Med. Genet. A.

[7] Loeys B., De Backer J., Van Acker P., Wettinck K., Pals G., Nuytinck L., Coucke P., De Paepe A. (2004). Comprehensive molecular screening of the FBN1 gene favors locus homogeneity of classical Marfan syndrome. Hum. Mutat..

[8] Landis B.J., Veldtman G.R., Ware S.M. (2017). Genotype-phenotype correlations in Marfan syndrome. Heart.

[9] Becerra-Muñoz V.M., Gómez-Doblas J.J., Porras-Martín C., Such-Martínez M., Crespo-Leiro M.G., Barriales-Villa R., de Teresa-Galván E., Jiménez-Navarro M., Cabrera-Bueno F. (2018). The importance of genotype-phenotype correlation in the clinical management of Marfan syndrome. Orphanet. J. Rare Dis..

[10] Xu S., Li L., Fu Y., Wang X., Sun H., Wang J., Han L., Wu Z., Liu Y., Zhu J. (2020). Increased frequency of FBN1 frameshift and nonsense mutations in Marfan syndrome patients with aortic dissection. Mol. Genet. Genom. Med..

[11] Faivre L., Collod-Beroud G., Loeys B.L., Child A., Binquet C., Gautier E., Callewaert B., Arbustini E., Mayer K., Arslan-Kirchner M. (2007). Effect of mutation type and location on clinical outcome in 1,013 probands with Marfan syndrome or related phenotypes and FBN1 mutations: An international study. Am. J. Hum. Genet..

[12] Faivre L., Collod-Beroud G., Callewaert B., Child A., Binquet C., Gautier E., Loeys B.L., Arbustini E., Mayer K., Arslan-Kirchner M. (2009). Clinical and mutation-type analysis from an international series of 198 probands with a pathogenic FBN1 exons 24-32 mutation. Eur. J. Hum. Genet..

[13] Wooderchak-Donahue W., VanSant-Webb C., Tvrdik T., Plant P., Lewis T., Stocks J., Raney J.A., Meyers L., Berg A., Rope A.F. (2015). Clinical utility of a next generation sequencing panel assay for Marfan and Marfan-like syndromes featuring aortopathy. Am. J. Med. Genet. A.

[14] Gentilini D., Oliveri A., Fazia T., Pini A., Marelli S., Bernardinelli L., Di Blasio A.M. (2019). NGS analysis in Marfan syndrome spectrum: Combination of rare and common genetic variants to improve genotype-phenotype correlation analysis. PLoS ONE.

[15] Kayhan G., Ergun M.A., Ergun S.G., Kula S., Percin F.E. (2018). Identification of Three Novel FBN1 Mutations and Their Phenotypic Relationship of Marfan Syndrome. Genet. Test Mol. Biomark..

[16] Torrado M., Maneiro E., Trujillo-Quintero J.P., Evangelista A., Mikhailov A.T., Monserrat L. (2018). A Novel Heterozygous Intronic Mutation in the FBN1 Gene Contributes to FBN1 RNA Missplicing Events in the Marfan Syndrome. Biomed. Res. Int..

[17] Oh M.R., Kim J.S., Beck N.S., Yoo H.W., Lee H.J., Kohsaka T., Jin D.K. (2000). Six novel mutations of the fibrillin-1 gene in Korean patients with Marfan syndrome. Pediatr. Int..

[18] Yoo E.H., Woo H., Ki C.S., Lee H.J., Kim D.K., Kang I.S., Park P., Sung K., Lee C.S., Chung T.Y. (2010). Clinical and genetic analysis of Korean patients with Marfan syndrome: Possible ethnic differences in clinical manifestation. Clin. Genet..

[19] Song Y.H., Kim G.H., Yoo H.W., Kim J.B. (2012). Novel de novo nonsense mutation of FBN1 gene in a patient with Marfan syndrome. J. Genet..

[20] Nam H.K., Nam M.H., Ha K.S., Rhie Y.J., Lee K.H. (2017). A Novel Fibrillin-1 Gene Mutation Leading to Marfan Syndrome in a Korean Girl. Ann. Clin. Lab. Sci..

[21] Seo G.H., Kim Y.M., Kang E., Kim G.H., Seo E.J., Lee B.H., Choi J.H., Yoo H.W. (2018). The phenotypic heterogeneity of patients with Marfan-related disorders and their variant spectrums. Medicine.

[22] Yoon S.H., Kong Y. (2021). Severe neonatal Marfan syndrome with a novel mutation in the intron of the FBN1 gene: A case report. Medicine.

[23] Nayak S.S., Schneeberger P.E., Patil S.J., Arun K.M., Suresh P.V., Kiran V.S., Siddaiah S., Maiya S., Venkatachalagupta S.K., Kausthubham N. (2021). Clinically relevant variants in a large cohort of Indian patients with Marfan syndrome and related disorders identified by next-generation sequencing. Sci. Rep..

[24] Richards S., Aziz N., Bale S., Bick D., Das S., Gastier-Foster J., Grody W.W., Hegde M., Lyon E., Spector E. (2015). Standards and guidelines for the interpretation of sequence variants: A joint consensus recommendation of the American College of Medical Genetics and Genomics and the Association for Molecular Pathology. Genet. Med..

[25] Desmet F.O., Hamroun D., Lalande M., Collod-Béroud G., Claustres M., Béroud C. (2009). Human Splicing Finder: An online bioinformatics tool to predict splicing signals. Nucleic Acids Res..

[26] Jaganathan K., Panagiotopoulou S.K., McRae J.F., Darbandi S.F., Knowles D., Li Y.I., Kosmicki J.A., Arbelaez J., Cui W., Schwartz G.B. (2019). Predicting Splicing from Primary Sequence with Deep Learning. Cell.

[27] Mannucci L., Luciano S., Salehi L.B., Gigante L., Conte C., Longo G., Ferradini V., Piumelli N., Brancati F., Ruvolo G. (2020). Mutation analysis of the FBN1 gene in a cohort of patients with Marfan Syndrome: A 10-year single center experience. Clin. Chim. Acta.

[28] Bombardieri E., Rohrbach M., Greutmann M., Matyas G., Weber R., Radulovic J., Boillat M.F., Linka A., De Pasquale G., Bonassin F. (2020). Marfan syndrome and related connective tissue disorders in the current era in Switzerland in 103 patients: Medical and surgical management and impact of genetic testing. Swiss Med. Wkly..

[29] Stengl R., Bors A., Ágg B., Pólos M., Matyas G., Molnár M.J., Fekete B., Csabán D., Andrikovics H., Merkely B. (2020). Optimising the mutation screening strategy in Marfan syndrome and identifying genotypes with more severe aortic involvement. Orphanet. J. Rare Dis..

[30] Overwater E., Marsili L., Baars M.J.H., Baas A.F., van de Beek I., Dulfer E., van Hagen J.M., Hilhorst-Hofstee Y., Kempers M., Krapels I.P. (2018). Results of next-generation sequencing gene panel diagnostics including copy-number variation analysis in 810 patients suspected of heritable thoracic aortic disorders. Hum. Mutat..

[31] Wang Z., Zhuang X., Chen B., Wen J., Peng F., Liu X., Wei M. (2020). 99-Case Study of Sporadic Aortic Dissection by Whole Exome Sequencing Indicated Novel Disease-Associated Genes and Variants in Chinese Population. Biomed. Res. Int..

[32] Li J., Yang L., Diao Y., Zhou L., Xin Y., Jiang L., Li R., Wang J., Duan W., Liu J. (2021). Genetic testing and clinical relevance of patients with thoracic aortic aneurysm and dissection in northwestern China. Mol. Genet. Genom. Med..

[33] Di C., Syafrizayanti, Zhang Q., Chen Y., Wang Y., Zhang X., Liu Y., Sun C., Zhang H., Hoheisel J.D. (2019). Function, clinical application, and strategies of Pre-mRNA splicing in cancer. Cell Death Differ..

[34] Wang Y., Sun Y., Liu M., Zhang X., Jiang T. (2019). Functional Characterization of Argininosuccinate Lyase Gene Variants by Mini-Gene Splicing Assay. Front. Genet..

[35] Wu Y., Sun H., He Y., Zhang H. (2021). A novel intron mutation in FBN-1 gene identified in a pregnant woman with Marfan syndrome. Hereditas.

[36] Wang W.J., Han P., Zheng J., Hu F.Y., Zhu Y., Xie J.S., Guo J., Zhang Z., Dong J., Zheng G.Y. (2013). Exon 47 skipping of fibrillin-1 leads preferentially to cardiovascular defects in patients with thoracic aortic aneurysms and dissections. J. Mol. Med..

[37] Wypasek E., Potaczek D.P., Hydzik M., Stapor R., Raczkowska-Muraszko M., Weiss J., Maugeri A., Undas A. (2018). Detection and a functional characterization of the novel FBN1 intronic mutation underlying Marfan syndrome: Case presentation. Clin. Chem. Lab. Med..

[38] Fusco C., Morlino S., Micale L., Ferraris A., Grammatico P., Castori M. (2019). Characterization of Two Novel Intronic Variants Affecting Splicing in FBN1-Related Disorders. Genes.

[39] Zeyer K.A., Reinhardt D.P. (2015). Engineered mutations in fibrillin-1 leading to Marfan syndrome act at the protein, cellular and organismal levels. Mutat. Res. Rev. Mutat. Res..

[40] Groth K.A., Gaustadnes M., Thorsen K., Østergaard J.R., Jensen U.B., Gravholt C.H., Andersen N.H. (2016). Difficulties in diagnosing Marfan syndrome using current FBN1 databases. Genet. Med..

